# Severe Hypoprothrombinemia Associated With Vitamin K Deficiency During Prolonged Cefazolin Therapy in a Hemodialysis Patient

**DOI:** 10.7759/cureus.110353

**Published:** 2026-06-06

**Authors:** Mari Morimoto, Yu Mihara, Munehiro Yoshida, Yasuto Sunahara, Nariko Ikemura, Kayo Kitamoto, Tetsuro Kusaba, Ryosuke Hamashima, Yoichi Yuki, Tohru Inaba, Keiichi Tamagaki

**Affiliations:** 1 Nephrology, Kyoto Prefectural University of Medicine, Kyoto, JPN; 2 Infection Control and Laboratory Medicine, Kyoto Prefectural University of Medicine, Kyoto, JPN

**Keywords:** cefazolin, coagulopathy, end-stage kidney disease, maintenance hemodialysis, vitamin k deficiency bleeding

## Abstract

This report presents a case of a dialysis patient who developed severe hypoprothrombinemia associated with vitamin K deficiency during prolonged cefazolin (CEZ) therapy. A 73-year-old man undergoing maintenance hemodialysis for end-stage kidney disease due to diabetic nephropathy was hospitalized for purulent tenosynovitis of the right ankle and treated with CEZ. During hospitalization, he had persistent anorexia and experienced recurrent gastrointestinal bleeding. On a non-dialysis day, he developed a subcutaneous hematoma around the arteriovenous fistula. Despite the absence of anticoagulant use, marked prolongation of prothrombin time and activated partial thromboplastin time was observed. Laboratory findings showed reduced activity of vitamin K-dependent coagulation factors and a markedly elevated protein induced by vitamin K absence or antagonist-II level, indicating vitamin K deficiency. Intravenous vitamin K administration and switching the antibiotic from CEZ to ampicillin resulted in rapid improvement of hypoprothrombinemia. This case suggests that in hemodialysis patients, hypoprothrombinemia may develop through cumulative effects of inadequate nutritional intake, prolonged antibiotic exposure, and bleeding-related coagulation factor depletion. Even in the absence of anticoagulant therapy, careful coagulation monitoring is warranted in hospitalized hemodialysis patients receiving prolonged antibiotic treatment.

## Introduction

Vitamin K is essential for the synthesis of vitamin K-dependent proteins (VKDPs), including coagulation factors II, VII, IX, and X. Vitamin K deficiency can lead to hypoprothrombinemia and bleeding complications [1]. Certain cephalosporins with specific side chains are known to interfere with vitamin K metabolism and, when combined with reduced dietary intake, may contribute to vitamin K deficiency. Although hypoprothrombinemia associated with cefazolin (CEZ) has been reported, clinically significant cases appear to be rare and have occurred mainly in patients with renal impairment, malnutrition, or prolonged antibiotic exposure [2]. Patients with end-stage kidney disease are particularly vulnerable, not primarily because of dialysis itself, but because reduced renal function can decrease the clearance of renally excreted antibiotics, and dietary restrictions for kidney failure may reduce vitamin K intake. Intercurrent bleeding events may further and cumulatively affect coagulation status [3]. Herein, we report a hemodialysis patient who developed severe hypoprothrombinemia through multifactorial and time-dependent mechanisms, in which prolonged inadequate intake, CEZ administration, and recurrent bleeding events appeared to synergistically contribute to the development of critical coagulopathy.

## Case presentation

A 73-year-old man with end-stage kidney disease secondary to diabetic nephropathy, who had been undergoing hemodialysis for 13 years, sprained his right ankle two weeks before admission. Despite conservative treatment, his pain persisted. Due to worsening pain in his right lower leg and a fever of up to 39.7°C, he was referred to our hospital. On examination, a 1.5 cm black necrotic lesion with surrounding erythema and tenderness was observed on the medial side of his right great toe. Blood tests showed stable hemoglobin levels (11.8 g/dL), indicating no progression of anemia. However, C-reactive protein was elevated (35.16 mg/dL), suggestive of an inflammatory response. Platelet count was 349,000/µL, fibrinogen was 453 g/dL, prothrombin time-international normalized ratio (PT-INR) was 1.51, and activated partial thromboplastin time (APTT) was prolonged to 45 seconds (Table 1).

**Table 1:** Laboratory data on admission

Parameter	Result	Reference
White blood cell	26.400 /μL	3.300-8.600/μL
Neutrophils	91.4%	45.2-68.8%
Lymphocytes	3.10%	26.8-43.8%
Red blood cell	4.02×10^6^ /μL	4.35-5.55 ×10⁶/μL
Hemoglobin	11.8 g/dL	13.7-16.8 g/dL
Platelet	34.9×10^4^ /μL	15.8-34.8 ×10⁴/μL
Prothrombin time	19.1 sec	9.0-13.0 sec
Prothrombin time-International ratio	1.51	0.80-1.20
Activated partial thromboplastin time	45 sec	23-40 sec
Fibrinogen	453 mg/dL	150-400 mg/dL
Total protein	6.4 g/dL	6.6-8.1 g/dL
Albumin	2.7 g/dL	4.1-5.1 g/dL
Aspartate transaminase	15 IU/L	13-30 U/L
Alanine transaminase	81 IU/L	10-42 U/L
Lactate dehydrogenase	313 IU/L	124-222 U/L
Alkaline phosphatase	81 IU/L	38-113 U/L
γ-glutamyltransferase	25 IU/L	13-64 U/L
Total bilirubin	0.47 mg/dL	0.4-1.5 mg/dL
Urea nitrogen	21.0 mg/dL	8-20 mg/dL
Creatinine	3.74 mg/dL	0.65-1.07 mg/dL
Sodium	135 mEq/L	138-148 mEq/L
Potassium	4.3 mEq/L	3.6-4.8 mEq/L
Chloride	95 mEq/L	101-108 mEq/L
Calcium	9.8 mg/dL	8.8-10.1 mg/dL
Phosphorus	1.3 mg/dL	2.7-4.6 mg/dL
Glucose	188 mg/dL	73-109 mg/dL
C-reactive protein	35.16 mg/dL	≤0.14 mg/dL

Although thrombocytopenia was not observed, hypoprothrombinemia was present. Computed tomography of the right ankle revealed fascial thickening and the presence of air within the soft tissue (Figure 1).

**Figure 1:**
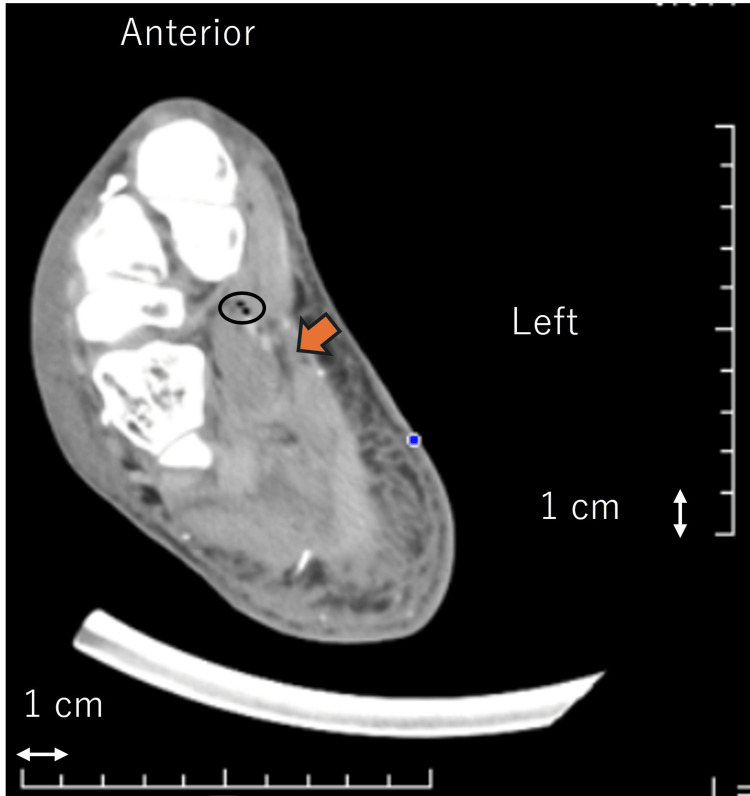
Axial computed tomography image of the right ankle Fascial thickening is indicated by the arrow, and soft-tissue air is indicated by the circle.

Surgical drainage was performed. The patient was diagnosed with purulent tenosynovitis and admitted to the hospital. The patient's past medical history included type 2 diabetes mellitus, hypertension, and hypothyroidism. There was no known history of coagulopathy based on the available medical records and clinical history. Coagulation tests had not been performed before admission; therefore, pre-admission baseline Prothrombin Time-International Normalized Ratio (PT-INR) and Activated Partial Thromboplastin Time (APTT) values were unavailable. He had no history of gastrointestinal endoscopy. His medications included nifedipine 20 mg, carvedilol 10 mg, ferric citrate hydrate 750 mg, and teneligliptin 40 mg. He was not taking anticoagulants or proton pump inhibitors. He was an occasional drinker and had a history of smoking (40 cigarettes/day for 20 years) but had quit.

Following surgical drainage, intravenous vancomycin was initiated. Before admission, heparin had been used as the anticoagulant during maintenance hemodialysis. However, after admission, it was replaced with nafamostat mesilate to prevent bleeding complications. Methicillin-sensitive *Staphylococcus aureus* was isolated from wound cultures; therefore, the antibiotic was switched to CEZ (2 g/day IV) on hospital day 6. Based on the presumed diagnosis of purulent tenosynovitis requiring prolonged antimicrobial therapy, an approximately 4-week treatment course was initially planned. Because local infectious findings persisted after surgical intervention, CEZ therapy was continued beyond the initial treatment period.

On hospital day 7, the patient developed acute anemia (Hb 4.7 g/dL) with a marked prolongation of PT-INR (4.18). Upper gastrointestinal endoscopy revealed a duodenal ulcer scar without an active bleeding source. Due to rapid progression of anemia and hemodynamic instability, continuous renal replacement therapy was initiated for two days. Because of severe anemia, allogeneic blood transfusions were administered before and during continuous renal replacement therapy. Notably, the PT-INR spontaneously improved to 1.15 on hospital day 11 without vitamin K supplementation. Surgical intervention and CEZ administration were continued; however, throughout hospitalization, the patient had persistent anorexia and was unable to maintain adequate oral intake, consuming only approximately 10-20% of the meals provided during hospitalization. Although 50% glucose and amino acid solutions were administered during dialysis, no vitamin supplementation was provided. By hospital day 35, his anemia had worsened, necessitating transfusions during dialysis. On hospital day 38, repeat upper gastrointestinal endoscopy revealed active bleeding from a duodenal ulcer, which was managed with endoscopic clipping. Vonoprazan fumarate was initiated, and the patient was placed on fasting. However, recurrent bleeding was observed on hospital day 40, requiring another hemostatic procedure.

On the following day (hospital day 41), a non-dialysis day, the patient developed swelling and pain of the arteriovenous fistula arm accompanied by a subcutaneous hematoma. Coagulation tests showed marked prolongation of PT-INR (16.91) and APTT (233.9 seconds) compared with values on admission (Figure 2).

**Figure 2:**
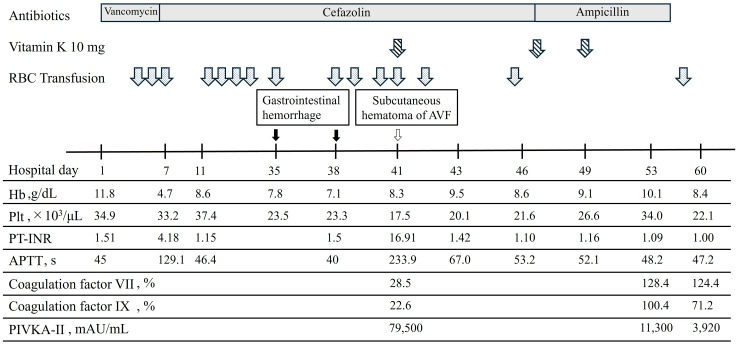
Clinical course of coagulopathy and anemia following transfusion and vitamin K2 therapy Monochrome downward arrows indicate red blood cell transfusion, and striped downward arrows indicate intravenous vitamin K administration. Major bleeding events and antibiotic exposure are shown above the hospital-day timeline. RBC, red blood cell; AVF, arteriovenous fistula; Hb, hemoglobin; Plt, platelet; PT, prothrombin time; INR, international normalized ratio; APTT, activated partial thromboplastin time; PIVKA-II, protein induced by vitamin K absence or antagonist II.

Vitamin K deficiency was suspected based on the concurrent prolongation of PT and APTT as well as the patient’s poor nutritional status. Alternative causes of combined PT and APTT prolongation were also evaluated. Liver dysfunction was considered unlikely because liver function tests at the time of peak coagulopathy were within normal limits (aspartate aminotransferase (AST)/alanine aminotransferase (ALT)/alkaline phosphatase (ALP) 11/1/47 U/L, lactate dehydrogenase (LDH) 133 U/L, γ-glutamyl transpeptidase (GTP) 14 U/L, and total bilirubin 0.14 mg/dL). Disseminated intravascular coagulation was also considered unlikely because there was no worsening infection, thrombocytopenia, or reduction in fibrinogen levels. In addition, cross-mixing studies later demonstrated a pattern consistent with coagulation factor deficiency rather than acquired coagulation factor inhibitors. Intravenous administration of vitamin K2 (10 mg) led to significant improvement within four hours (PT-INR 2.67, APTT 102.3 seconds). Laboratory tests revealed deficiencies in multiple vitamin K-dependent coagulation factors (Factor II: 20.1%, Factor VII: 28.5%, Factor IX: 22.6%, Factor X: 11.2%) and a markedly elevated protein induced by vitamin K absence or antagonist-II (PIVKA-II, an abnormal prothrombin produced during vitamin K deficiency) level (70,500 mAU/mL, reference < 39 mAU/mL), confirming the diagnosis.

Because coagulation parameters were measured only at the time points shown in Figure 2, the exact timing of the initial PT-INR prolongation after CEZ initiation could not be determined. Given the potential role of CEZ in inhibiting the vitamin K cycle, the antibiotic was switched to ampicillin on hospital day 47. Following three doses of intravenous vitamin K2, PT-INR normalized by hospital day 46 (1.10), and levels of vitamin K-dependent coagulation factors returned to normal by hospital day 52 (Factor II: 86.5%, Factor VII: 128.4%, Factor IX: 100.4%, Factor X: 61.2%). PIVKA-II levels had decreased to 1,530 mAU/mL by hospital day 63. Following the development of gastrointestinal bleeding and subsequent subcutaneous hematoma of the arteriovenous fistula arm, allogeneic blood transfusions were administered as clinically indicated according to bleeding events and worsening anemia during the clinical course. No further bleeding episodes occurred. The right ankle tenosynovitis improved with continued surgical management and wound care. The patient was transferred to a rehabilitation facility on hospital day 80.

## Discussion

This case suggests that clinically significant coagulopathy in hemodialysis patients may reflect the cumulative and time-dependent interaction of multiple factors rather than a single drug effect. Vitamin K functions as a cofactor for γ-glutamyl carboxylase, which activates vitamin K-dependent proteins (VKDPs) and is involved in physiological processes including blood coagulation, inhibition of vascular calcification, bone metabolism, and regulation of cell growth [1-3]. Seventeen VKDPs have been identified in the human body, among which coagulation factors II, VII, IX, and X, as well as protein C and protein S, play an important role in hemostasis. Vitamin K exists in two main forms: vitamin K1 (phylloquinone) and vitamin K2 (menaquinone). Phylloquinone is predominantly derived from dietary sources, including green leafy vegetables and soy products, whereas menaquinone is found in dairy products and poultry and is also synthesized by intestinal microbiota. The majority of dietary vitamin K intake consists of phylloquinone, which is metabolized into menaquinone and exerts various physiological effects [4].

Vitamin K deficiency leads to reduced activation of VKDPs and is characterized by decreased levels of vitamin K-dependent coagulation factors (II, VII, IX, and X) and elevated levels of PIVKA-II [2,5,6]. Because factor VII has the shortest half-life among these coagulation factors, prolongation of PT-INR is typically the earliest laboratory indicator of vitamin K deficiency, and severe deficiency may also prolong APTT. Hypoprothrombinemia due to vitamin K deficiency generally improves within approximately 48 hours following vitamin K administration [7-9]. In routine clinical practice, direct measurement of vitamin K levels is uncommon; therefore, diagnosis is based on characteristic coagulation abnormalities, elevated PIVKA-II levels, and rapid correction after vitamin K supplementation.

The etiology of vitamin K deficiency is multifactorial and includes reduced dietary intake, impaired intestinal absorption, decreased intestinal microbiota, and disruption of the vitamin K cycle. Vitamin K deficiency rarely results from a single cause and more commonly develops through the cumulative effects of multiple contributing factors. Although the daily requirement for vitamin K is relatively low [1,10,11], patients with chronic kidney disease and those on dialysis are particularly vulnerable because of dietary restrictions, impaired nutritional intake during hospitalization, and altered drug pharmacokinetics [12,13].

Certain antibiotics are known to contribute to vitamin K deficiency by suppressing intestinal microbiota and interfering with the vitamin K cycle [1]. Cephalosporins containing an N-methylthiotetrazole (NMTT) side chain, such as cefmetazole and cefoperazone, directly inhibit vitamin K epoxide reductase, resulting in warfarin-like effects [8,14,15]. CEZ, although lacking an NMTT side chain, contains a methyl-thiadiazole-thiol group and has been reported to exert a weaker inhibitory effect on the vitamin K cycle [7,16-18]. Clinically significant hypoprothrombinemia associated with CEZ is uncommon in isolation but has been reported predominantly in patients with renal impairment or malnutrition, suggesting that additional host-related factors are necessary for its manifestation. Therefore, although CEZ is not classically regarded as a high-risk cephalosporin for hypoprothrombinemia compared with NMTT-containing cephalosporins, prolonged CEZ therapy should be used with caution in dialysis patients with poor nutritional intake or other risk factors for vitamin K deficiency. In such high-risk patients, periodic monitoring of coagulation parameters, particularly PT-INR, may be considered during prolonged CEZ therapy.

The present case illustrates that severe hypoprothrombinemia in hemodialysis patients may develop through dynamic and multifactorial processes rather than a single causative factor. Notably, a marked prolongation of PT-INR was observed early in hospitalization, coinciding with acute anemia on hospital day 7, and subsequently improved without vitamin K supplementation (Figure 2). These transient coagulation abnormalities may be attributable to acute consumption of coagulation factors during bleeding, a phenomenon known as consumptive coagulopathy [19,20].

As hospitalization progressed, the patient experienced persistent anorexia and inadequate oral intake, while receiving prolonged CEZ therapy in the setting of impaired renal clearance. These conditions likely resulted in cumulative depletion of vitamin K stores. Under this compromised baseline, recurrent gastrointestinal bleeding episodes may have further reduced circulating coagulation factors, ultimately precipitating critical hypoprothrombinemia and arteriovenous fistula bleeding. The dramatic and rapid improvement in coagulation parameters following vitamin K administration, along with markedly elevated PIVKA-II levels, supports the coexistence of progressive vitamin K deficiency and superimposed coagulation factor loss. Multivitamin preparations used in total parenteral nutrition generally provide sufficient vitamin K; however, in this case, vitamin supplementation was not provided despite prolonged inadequate oral intake. Earlier recognition of nutritional insufficiency and proactive vitamin supplementation may have mitigated the progression to severe coagulopathy.

## Conclusions

This case highlights that coagulation abnormalities in hemodialysis patients can fluctuate and temporarily improve, potentially masking an underlying progressive coagulopathy. Prolonged inadequate nutritional intake and antibiotic exposure, when combined with intercurrent bleeding events, may synergistically precipitate critical bleeding even in the absence of anticoagulant therapy. Clinicians should maintain a high index of suspicion for evolving vitamin K deficiency during prolonged hospitalization and consider nutritional and coagulation monitoring, as well as timely vitamin supplementation, in dialysis patients receiving extended antibiotic therapy.
